# *Maullinia braseltonii* sp. nov. (Rhizaria, Phytomyxea, Phagomyxida): A Cyst-forming Parasite of the Bull Kelp *Durvillaea* spp. (Stramenopila, Phaeophyceae, Fucales)

**DOI:** 10.1016/j.protis.2017.07.001

**Published:** 2017-07-13

**Authors:** Pedro Murúa, Franz Goecke, Renato Westermeier, Pieter van West, Frithjof C. Küpper, Sigrid Neuhauser

**Affiliations:** a Oceanlab, School of Biological Sciences, University of Aberdeen, Main street, Newburgh, AB41 6AA, United Kingdom; b Aberdeen Oomycete Laboratory, International Centre for Aquaculture Research and Development, University of Aberdeen, Foresterhill, Aberdeen, AB25 2ZD, United Kingdom; c The Scottish Association for Marine Science, Scottish Marine Institute, Culture Collection for Algae and Protozoa, Oban, Argyll, PA37 1QA, United Kingdom; d Department of Plant and Environmental Science (IPV), Norwegian University of Life Sciences (NMBU), Ås, Norway; e Laboratorio de Macroalgas, Instituto de Acuicultura, Universidad Austral de Chile, Sede Puerto Montt. PO box 1327, Puerto Montt, Chile; f Institute of Microbiology, University of Innsbruck, Innsbruck, Tyrol, Austria

**Keywords:** plasmodiophorids, brown algae, galls, rDNA, resting spores

## Abstract

Phytomyxea are obligate endoparasites of angiosperm plants and Stramenopiles characterised by a complex life cycle. Here *Maullinia braseltonii* sp. nov., an obligate parasite infecting the bull kelp *Durvillaea* (Phaeophyceae, Fucales) from the South-Eastern Pacific (Central Chile and Chiloe Island) and South-Western Atlantic (Falkland Islands, UK) is described. *M. braseltonii* causes distinct hypertrophies (galls) on the host thalli making it easily identifiable in the field. Sequence comparisons based on the partial 18S and the partial 18S-5.8S-28S regions confirmed its placement within the order Phagomyxida (Phytomyxea, Rhizaria), as a sister species of the marine parasite *Maullinia ectocarpii*, which is also a parasite of brown algae. The development of resting spores in *M. braseltonii* is described by light and electron microscopy and confirmed by FISH experiments, which visually showed the differential expression of the 28S non-coding gene, strongly in early plasmodia and weakly in late cysts. *M. braseltonii* is, so far, the only phytomyxean parasite of brown algae for which the formation of resting spores has been reported, and which is widely distributed in *Durvillaea* stocks from the Southeastern Pacific and Southwestern Atlantic.

## Introduction

*Durvillaea* is a genus within the Phaeophyceae (Stramenopiles, Fucales) that comprises 6 species, all of them living in the intertidal or subtidal rocky habitats in the southern hemisphere, mainly with a sub-antarctic distribution (Fraser et al. 2010b; Guiry and Guiry 2016). Particularly in Chile there are two genetically different *Durvillaea* entities: the provisionally called “*continental clade”* (30°S to 44°S), and *Durvillaea antarctica* “*sub-antarctic clade*” from 49°S southward, which is also present in the Falkland Islands, several sub-antarctic islands and New Zealand (Fraser et al. 2010a). *Durvillaea* species commonly known as bull kelps are large, eye-catching organisms in marine habitats where they fulfil key ecological roles for coastal marine communities (Castilla and Bustamante 1989; Westermeier et al. 1994). As primary producers, bull kelp forests play a vital role in marine trophic networks, function as microhabitat, nursery and substratum for a number of organisms. Because of their size they are also significant for the understory flora and fauna, providing refuge for numerous organisms (Taylor and Schiel 2005). Bull kelps also play an important role in spreading the macroscopic and microscopic lifeforms associated with them (López et al. 2017). After individual kelps get detached they can – under the right circumstances – stay alive for a prolonged period of time (overall around 1 month) and travel over hundreds of kilometres (Fraser et al. 2011; Graiff et al. 2013). This increases their potential for their own dispersal along with the dispersal of their associated flora, fauna and likely their microbiome. Some countries have developed bull-kelp fishery/aquaculture. For such countries *Durvillaea* species are important in local economies and therefore harvested at different intensities (Castilla et al. 2007). *Durvillaea* was an important food element ca. 14,000 years ago for the earliest humans arriving in South America (Dillehay et al. 2008), and it is still consumed in Chile but also used in gourmet cuisine in Europe and Asia. In addition, both Chile and New Zealand have landing records for *Durvillaea* for alginate manufacturing (Schiel and Nelson 1990; Sernapesca 2015).

Large brown algae have been reported to be hosts of many pathogens, including viruses, bacteria and eukaryotic pathogens such as oomycetes and fungi (Müller et al. 1999; Sawabe et al. 1998; Schroeder 2015). The best known – and the only reported brown alga – parasite of *Durvillaea* spp. is *Herpodiscus durvilleae* (Sphacelariales), which causes lesions of the fronds (South 1974). Overall, it is unknown to which extent pathogens are involved as ecological drivers in kelp communities and seaweeds in general, but their influence on metabolism, reproduction, productivity and mortality in natural stocks is becoming increasingly evident (Gachon et al. 2010; Goecke et al. 2010; Neuhauser et al. 2011). With the increasing production and economic importance of seaweed mariculture such parasites have gained more awareness because disease outbreaks and subsequent massive mortalities may cause significant losses (Loureiro et al. 2015). Yet, our knowledge in the field is rather limited, and largely based on circumstantial or even accidental findings.

Phytomyxea (Rhizaria, Endomyxa/Phytorhiza) are obligate parasites of plants and Stramenopiles (Neuhauser et al. 2014). They are characterised by a complex life cycle with two spore-forming phases: the sporangial (primary plasmodium, primary zoospores) and the sporogenic stages (secondary plasmodium, resting spores) (Bulman and Braselton 2014). Phytomyxea are divided in two orders, Plasmodiophorida, parasites of land plants and oomycetes and Phagomyxida, infecting marine algae and seagrasses (Neuhauser et al. 2014). To date the only described phytomyxid parasite of brown algae is the phagomyxid *Maullinia ectocarpii,* a parasite isolated from *Ectocarpus siliculosus* (Ectocarpales). In the laboratory, it also infected ten additional brown algal species from four different orders, including kelp gametophytes (Maier et al. 2000). The species we pursue to describe is sister of *M. ectocarpii* and parasitic of *Durvillaea* spp. (Fucales) in Chile and in the Falkland Islands (Supplementary Material Fig. S1).

Over the last 40 years, galled bull kelp individuals have been reported all across the Southern hemisphere, in natural populations from Chile, Australia and Macquarie Island (Aguilera et al. 1988; Jahnke 1978; Ricker 1987). In some cases histological observations suggested that these symptoms may be linked with a phytomyxid pathogen (formerly known as plasmodiophorids). Goecke et al. (2012) managed to produce the first partial 18S rRNA phylogeny from this parasite found in Central and Southern Chile and established that the *Durvillaea* pathogen is related to *Maullinia ectocarpii*. They also found evidence of resting spores. However, both phylogenetic and microscopic evidence was not enough to support the conclusion that the *Durvillaea* pathogen may be a new species. In this study we formally describe the causal agent responsible for gall formations in the bull kelp species *D. antarctica* (sub-antarctic clade) and *Durvillaea sp.* (continental clade).

## Results

### Morphology and Development of the Parasite

**Figure 1:**
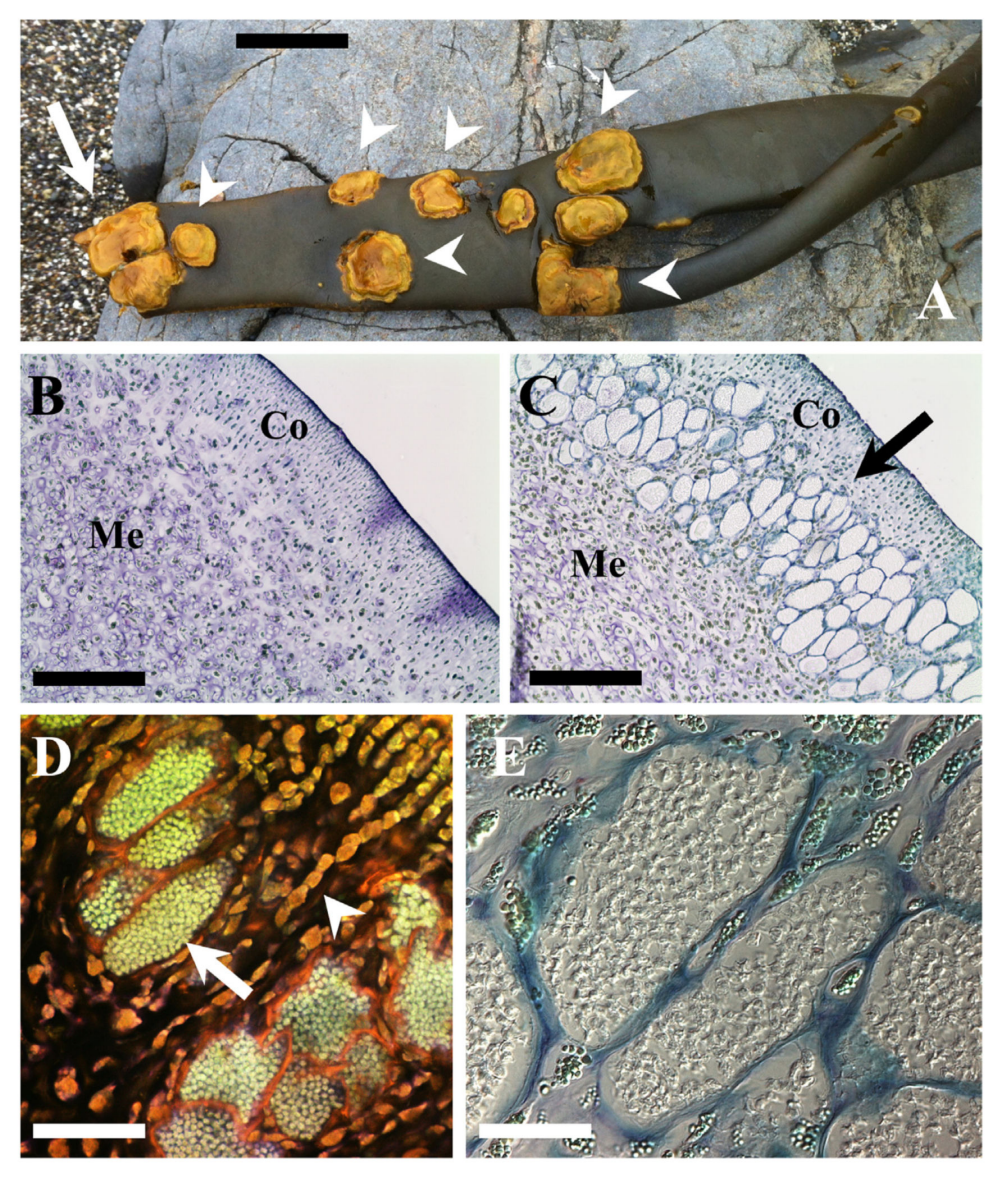
*Maullinia braseltonii* sp. nov. infecting the bull kelp *Durvillaea antarctica.* **A**: Galls (white arrrowheads) on a *Durvillaea antarctica* blade that was found washed at the beach at Mar Brava. Arrow indicates that the area where the frond ruptured. It shows a dense cluster of galls. Scale bar: 12 cm. **B**–**C**: Overview of cross sections of healthy (**B**) and infected (**C**) *D. antarctica* thalli. Co: cortex; Me: medulla. Arrow points at infected cells. Scale bars: 200 μm. **D:** Propidium iodine staining showing the extent of the hypertrophies. Normal sized, uninfected *D. antarctica* cells (arrowhead) and hypertrophied *D. antarctica* cells filled with resting spores (arrow). Scale bar: 100 μm. **E**: Host cell filled with young resting spores (Light microscopy - DIC). Scale bar: 20 μm.

Macroscopic symptoms: Galls were formed on the fronds of *Durvillaea*. The galls were brownish-yellow in contrast to the typical dark colouration of the host (Fig. 1A). Galls had an irregular surface in contrast of the smooth thallus, may appear solitarily or in patches (Fig. 1A) and are circular or ellipsoid with a size of up to 10 × 7 cm in diameter but can be as small as 1 × 1 cm (Table 1; Supplementary Material Table S1). These galls were mostly observed on the fronds but sometimes could be spotted on the bull kelp stipe. In our sampling expeditions, macroscopic symptoms were recognized predominantly in adults, and were mostly absent within the populations in periods of major bull kelp recruitment (as in summer 2015), except for diseased old drifted individuals that were present throughout all the year. On the other hand, when adults were more common, such as in winter 2015/2016 in Mar Brava, ca. 40% of the population, showing a density of 3–9 individuals m^−2^, were infected. Kelps that were bearing galls were often seen washed onto the beach across the study areas (fresh and dehydrated along the shore), and they seemed to have ruptured in an area with a cluster of galls (Fig. 1A). This is in accordance with the observation that the galls appear to make the thallus harder and less elastic in the affected areas.

**Figure 2:**
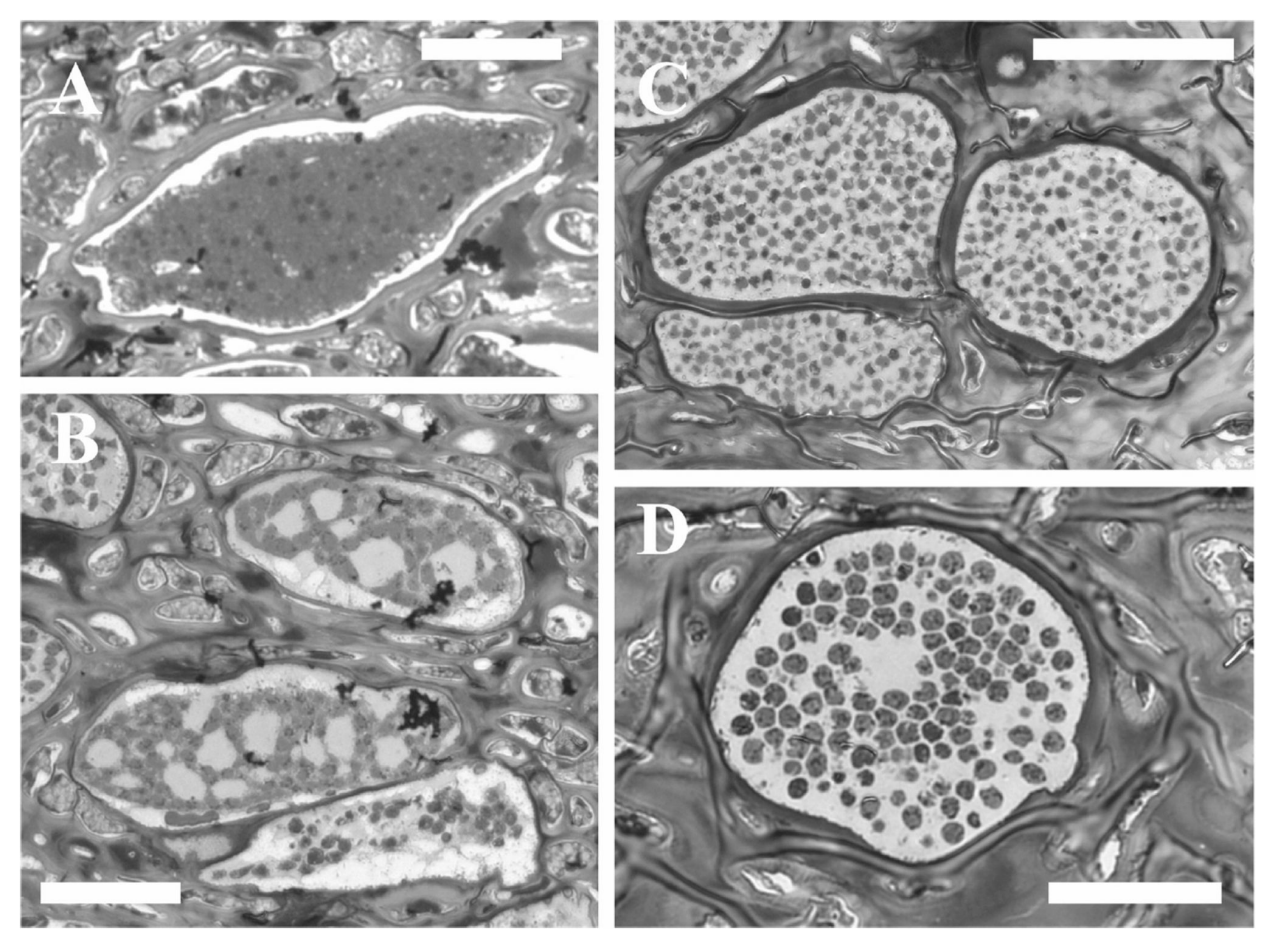
Light microscopy of semi-thin sections of *M. braseltonii*. **A:** Multinucleate plasmodium. Scale bar: 25 μm. **B:** Lobose plasmodia which are cleaving into resting spores. Scale bar: 25 μm. **C:** Still irregular, young resting spores. Scale bar: 40 μm. **D:** Host cell filled with sub-globose to broad elliptic, slightly irregular resting spores. Scale bar: 20 μm.

**Figure 3:**
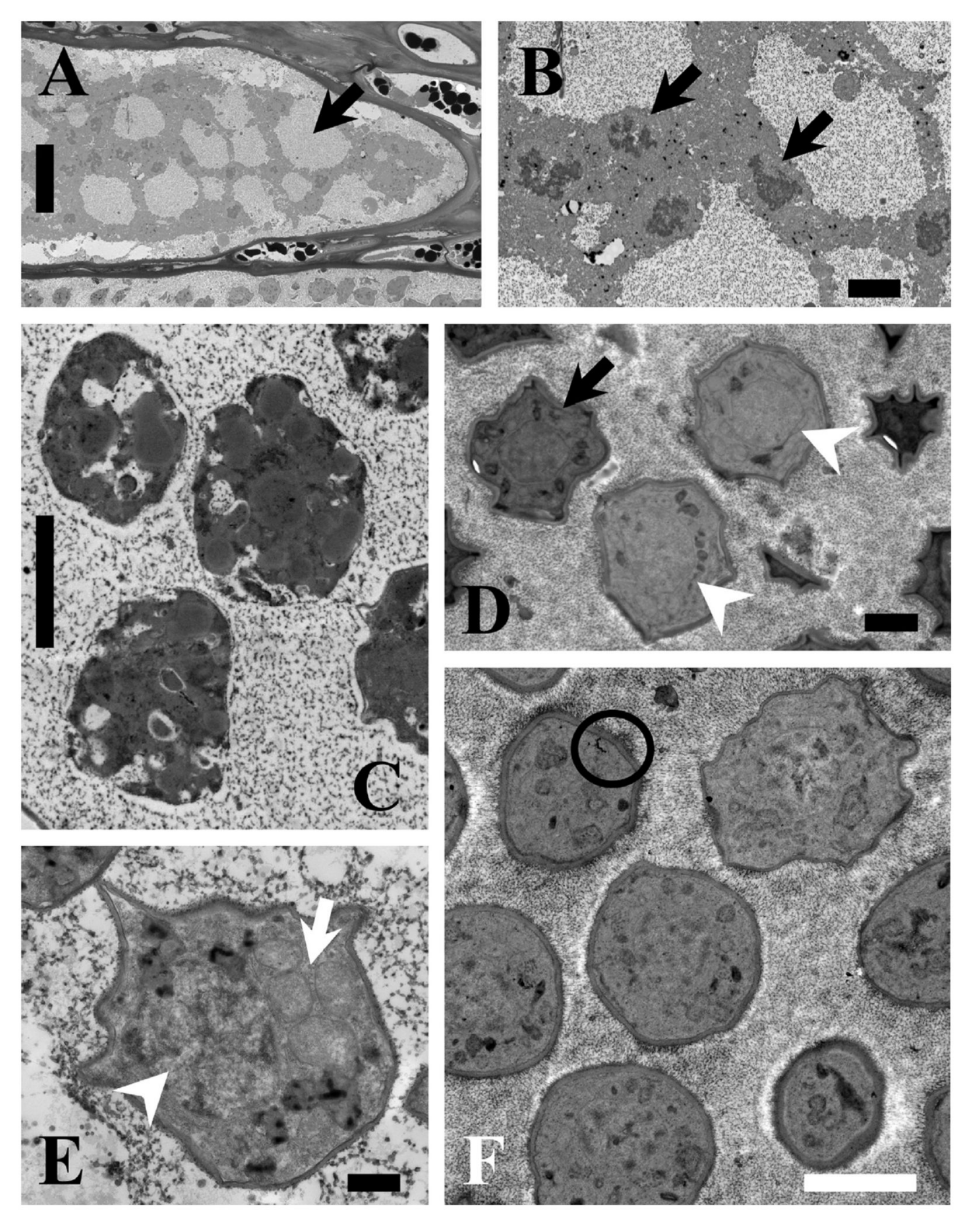
TEM images of *M. braseltonii*. **A:** Cell filled with a lobose plasmodium (Arrow). Scale bar: 20 μm. **B:** Details of the multiple, dividing nuclei (arrows) distinctive for growing plasmodia. Scale bar: 2 μm. **C:** Plasmodium cleaving into resting spores. The individual cells are already organised but the cell wall is not yet visible. Scale bar: 2 μm. **D**: Maturing resting spores, which are still slightly irregular in shape (arrowheads), but the multi-layered cell wall is already visible in some of them (arrow). Scale bar: 1 μm. **E:** Detail showing the nucleus (arrowhead) and mitochondria (arrow) of a developing resting spore. 500 nm. **F**: Ripe resting spores with the characteristic multi-layered cell wall (circle). Scale bar: 2 μm.

**Figure 4:**
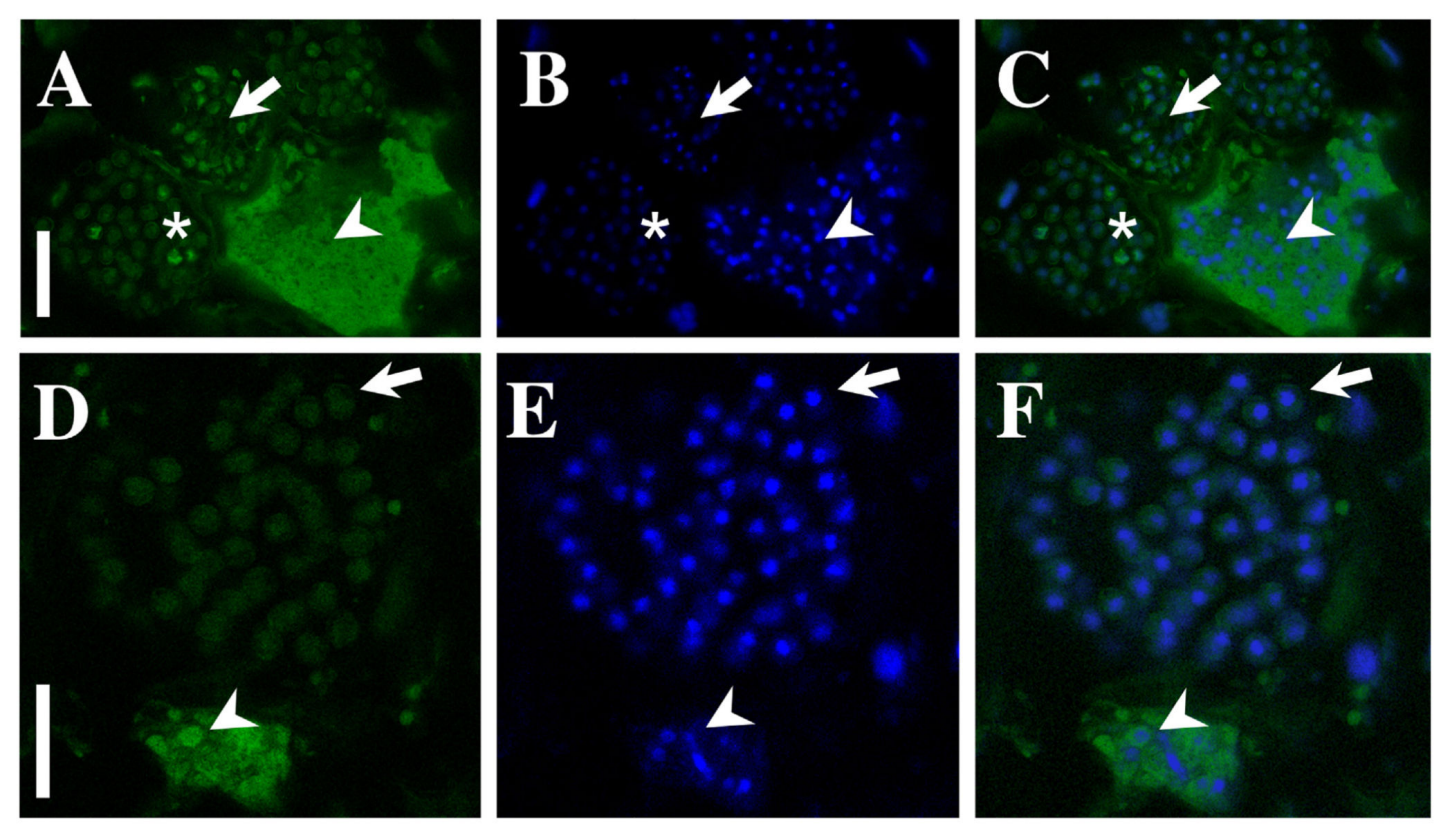
FISH-staining of the 28S rDNA region of *M. braseltonii*. **A–C**: FISH signal (**A**), DAPI staining (**B**) and an overlay of both channels (**C**) showing a multinucleate plasmodium (arrowhead) and developing resting spores (arrow). The brighter colouration of the plasmodium indicates a high physiological activity in the growing plasmodia. The DNA is arranged differently in plasmodia and the spores as DAPI staining shows longish-irregular, dividing nuclei in the plasmodium (arrowhead) and a condensed nucleus in the resting spores (arrow). The asterisk is highlighting a just forming resting spore which can be identified by the strong FISH-signal around the edges and the not well visible nuclei in DAPI staining. Scale bar = 20 μm. **D–F**: FISH signal (**D**), DAPI staining (**E**) and an overlay of both channels (**F**) showing a multinucleate plasmodium (arrowhead) and developing resting spores (arrow). The plasmodium is very mature as the ribosomes aggregate in the form of the resting spores (arrowhead) while the resting spores in this image are fully mature as inferred from the shape of the nucleus and the weaker ribosomal FISH signal (arrow). Scale bar = 15 μm.

Microscopic characteristics: In cross sections of infected *Durvillaea* fronds, the disease was restricted to the region between the cortex and the medulla tissue (Fig. 1B-C). We have not seen any parasite structures in the host meristoderm or in the medulla tissue. Infected cells were enlarged and filled with the parasite (Fig. 1C-E) which causes the macroscopically visible hypertrophies. One individual lobose multicellular plasmodium could be observed in each hypertrophied cell. The plasmodia were variable in size and shape and could reach sizes of up to 119 × 73 µm (Table 1; Supplementary Material Table S2) in comparison to ca. 15 × 10 µm of the healthy subcortical cells. The degree of hypertrophy was depending on the plasmodia development (Fig. 2A-D). Initially plasmodia filled the complete host cell (Fig. 2A) and developed a lobose structure (Figs 2B; 3A). In the growing plasmodium nuclei divided (Fig. 3B) until the plasmodia cleaved into resting spores (Figs 2C-D; 3C, 4A-F).

The resting spores were initially somewhat irregular in shape (Figs 2C; 3D-F). Resting spores were 3.2 ± 0.28 µm × 2.7 ± 0.30 µm in size across the broadest planes (Table 1, Supplementary Material Table S3). The spores were sub-globose to broad elliptic (length/width quotient: 1.2 ± 0.13), and thick-walled without colouration (Figs 1E; 3F). Each resting spore contained one nucleus (Figs 3E; 4B-C, E-F). All resting spores within an infected cell were formed from one individual plasmodium. During the development of the resting spores the cytoplasm started to concentrate around the nucleus (Figs 3C; 4A-F). Then the thick, multi-layered cell wall started to form. Initially the resting spores displayed an irregular shape (Fig. 3D-F) which once the cell matures got more regular. However, the spores kept a slightly asymmetrical shape and did not form near perfect round spores as observed in other phytomyxid species. The resting spores were not arranged into any form of cystosorus. The cell wall of the resting spores was three-layered (Fig. 3E).

### Phylogenetic Analyses

**Figure 5:**
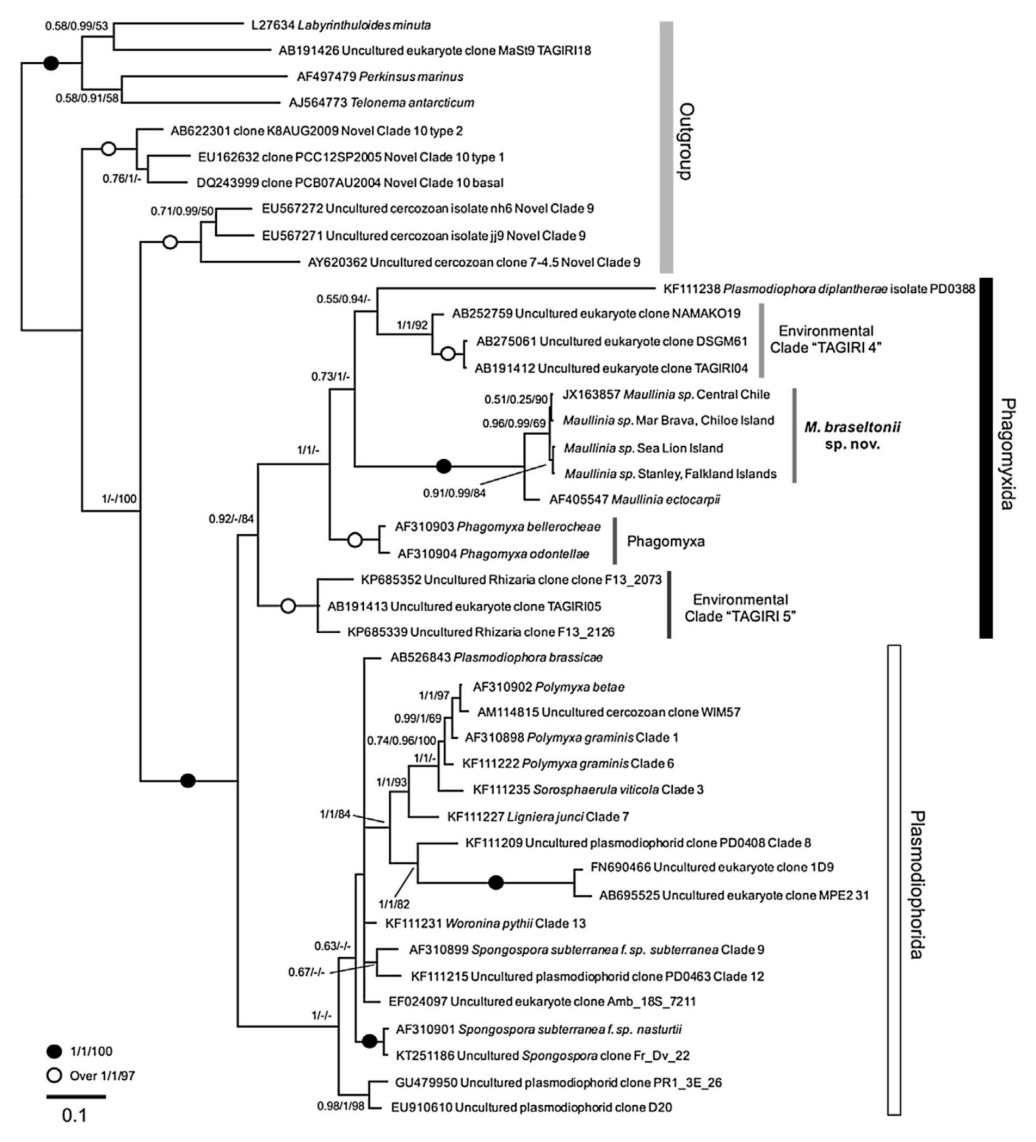
Bayesian analyses of *M. braseltonii* and all available phytomyxid sequences (from known species and environmental 18S rDNA clades). The tree contains a total of 43 sequences and 1002 positions. Support values given are posterior probabilities (MrBayes)/χ^2^ support values (PhyML)/bootstrap support (RAxML). MrBayes settings: chain length 1.000.000, subsample frequency 1.000, burn in of 10%. The scale bar indicates the number of substitutions per site. The accompanying PhyML (Supplementary Fig. 2) and RAxML (Supplementary Fig. 3) trees are provided in the Supplement. The genus *Maullinia* is well supported and *M. braseltonii* and *M. ectocarpii* form distinct branches on the tree. Phytomyxea (Phagomyxida and Plasmodiophorida) form a well-supported, monophyletic clade.

**Figure 6:**
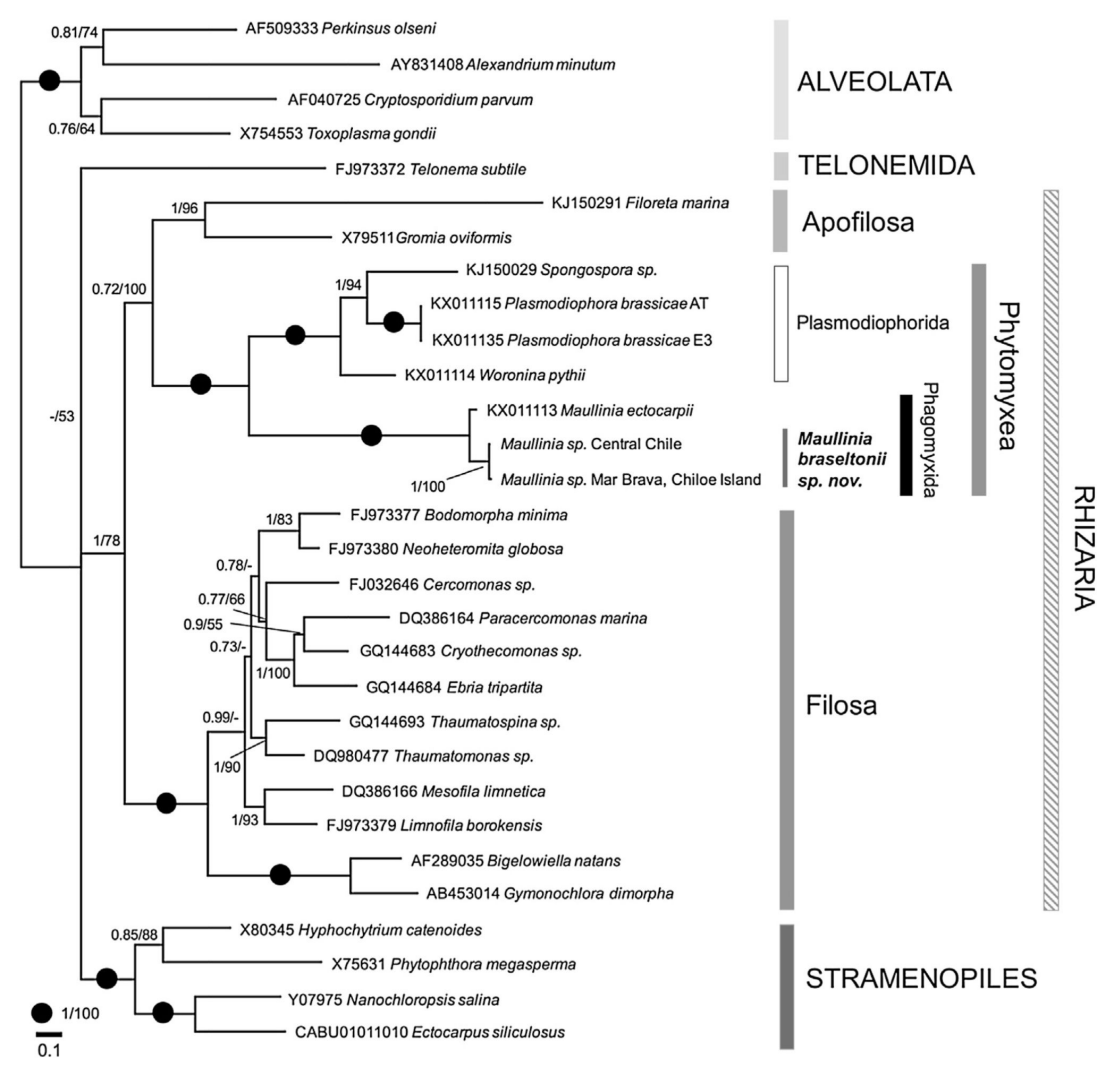
Bayesian analyses of *M. braseltonii* based on partial -18S-5.8S-partial-28S rDNA regions. 30 sequences and 1767 positions (ITS1, ITS2 were excluded from the analyses). Support values given are posterior probabilities (MrBayes)/bootstrap values (RAxML). The tree presented here was generated using MrBayes, chain length 1.000.000, subsample frequency 1.000, burn in of 10%. The scale bar indicates the number of substitutions per site. RAxML tree is provided as Supplementary Figure 4. The genus *Maullinia* is well supported and *M. braseltonii* and *M. ectocarpii* form distinct branches on the tree.

Based on 18S rDNA (Fig. 5) and 18S-5.8S-28S rDNA (Fig. 6) phylogenies the genus *Maullinia* formed a well-supported, distinct clade with all phylogenetic methods used. 18S rDNA trees include a comprehensive selection of phytomyxid taxa (plasmodiophorid and phagomyxid). The different phytomyxid taxa are well resolved and the tree topology conforms with other phylogenies of the group. In these trees *M. ectocarpii* and *M. braseltonii* consistently form separate clades, with high support values on different hierarchical nodes of the tree (Fig. 5). The differences in the 701 bp region of the 18S used is 3–11 bp (99% similarity) between the isolates from the Chilean Pacific coast and the sequences of the two isolates from the Atlantic coast. The differences between *M. ectocarpii* and *M. braseltonii* are 27–37 bp (95–96% similarity). When analysing the taxonomically more broadly sampled partial 18S-5.8S- partial 28S trees, *M. ectocarpii* and *M. braseltonii* form a well-supported clade within the phytomyxids, but the two species are well separated with high support values (Fig. 6). In the 1201 bp region of the 18S-5.8S-28S alignment differences between *M. braseltonii* from Mar Brava (Southern Chile) and Coliumo (Central Chile) are 9 bp (99% sequence similarity) while the difference between *M. ectocarpii* and *M. braseltonii* is 150 bp (sequence similarity 88%). Sequences were deposited in Genbank with accession numbers KY652636–KY652640.

### Diagnoses

#### Genus *Maullinia* I. Maier, E. R. Parodi, R. Westermeier et D. G. Müller

Addition to Maier et al.: Resting spores can be formed. Resting spores are sub-globose to broad elliptic and thick-walled without colouration.

#### *Maullinia braseltonii* sp. nov. P. Murúa, F. Goecke et S. Neuhauser

Characters of the species: Infecting *Durvillaea* spp., causing large yellowish hypertrophies on the fronds. Forming resting spores located in the area between the host cortex and medullar tissue. Spores are 3.2 µm +/− 0.28 µm in length and 2.69 µm +/− 0.30 µm in diameter. Resting spores are formed in masses from one plasmodium. Multinucleate plasmodia (27–119 µm) fill the host cells which are hypertrophied (ca. by factor 5-10). Zoospores and primary sporangia have not been observed.

Etymology: the species epithet refers to James P. Braselton, a well renowned researcher in the field of phytomyxid morphology and taxonomy to whom we intend to dedicate the species.

Hapantotype: Permanent microslides and galls fixed in 4% PFA of *Durvillaea* prepared from material collected at Mar Brava (Chiloe Island) in 2015 were deposited at the collection of the Natural History Museum in London (voucher numbers NHMUK 2017.2.9.1–NHMUK 2017.2.9.4).

Parahapantotype: Same fixed material as the hapantotype, deposited at the mycological collection of the University of Innsbruck (vouchers IB2017-0001/0002). DNA extracts are available from the authors. A further specimen from Sea Lion Island, collected on Dec. 12, 2013 (121213-1 FCK) was deposited in the Herbarium of the University of Aberdeen (ABDUK:001946).

## Discussion

Phytomyxea are divided into the marine Phagomyxida, which are parasites of marine angiosperms, brown algae and diatoms, and Plasmodiophorida, which are parasites of land plants and oomycetes (Neuhauser et al. 2014). All of them share common characteristics within their complex bi-phasic life cycle, such as cruciform nuclear division, zoospores with two anterior flagella of unequal size, the formation of multinucleate plasmodia and in most cases the formation of resting spores. Traditionally, a combination of resting-spore arrangement and ultrastructure was used to delimitate species. However, the description of the brown algal parasite *M. ectocarpii* (Maier et al. 2000) and the diatom parasites *Phagomyxa bellerochae* and *Phagomyxa odontellae* (Schnepf et al. 2000) along with rDNA-based phylogenetic studies challenged this species concept (Bulman et al. 2001; Neuhauser et al. 2014). The aforementioned species lack the formation of resting spores and the presence of resting-spore forming species within the Phagomyxida was only reported a few years ago (Goecke et al. 2012; Neuhauser et al. 2014). The parasite of brown algae described here is the first species parasitic on a marine stramenopile host where resting spores are observed. The resting spores are not arranged in any form of a sporosorus, but the way the resting spores are formed morphologically strongly resembles the formation of resting spores in other phytomyxid species (Bulman and Braselton 2014). The multinucleate plasmodia cleave into individual resting spores that are surrounded by the characteristic multi-layered cell wall upon maturity (Fig. 3).

During our examinations we only observed non-cruciform sporogenic plasmodia. The plasmodia and resting spores of *M. braseltonii* are located in between the cortical and the medulla cells of *Durvillaea* spp. Such a restricted localisation of the sporogenic phase of the life cycle is found in a number of phytomyxid species, such as *Plasmodiophora brassicae* or *Spongospora subterranea* where sporogenic plasmodia are located in the plant cortical cells (Bulman and Braselton 2014) and sporangial plasmodia are restricted to the epidermal cells. Spatial separation of life-cycle stages is even more prominent in *Sorosphaerula veronicae*, where sporogenic plasmodia are restricted to the shoot and sporangial plasmodia to the roots (Miller 1958), while other species such as *Polymyxa graminis* or *Ligniera junci* show no spatial separation of the different life-cycle stages. We have not found anything that resembled sporangial plasmodia in our samples. Therefore, it is possible that such a spatial separation of the two parts of the life cycle exists in *Maullinia* spp. as well. This hypothesis of a spatially segregated life cycle is further supported by the observations made on *M. ectocarpii*: the sporangial part of the life cycle was found in a filamentous brown alga (*Ectocarpus* spp.) but can also be seen in microthalli of heteromorphic brown algae (e.g. the giant kelp *Macrocystis pyrifera*), indicating that the sporangial part might be depending on a different type of host cell or tissue to be initiated. Since the reasons for how each sporangial and sporogenic developing program is triggered is not understood in phytomyxids in general, this aspect of the life cycle remains to be addressed in the future for *Maullinia* species.

So far, in *M. ectocapii* only the sporangial stage of the life cycle has been identified despite efforts to find the sporogenic phase by Parodi et al. (2010), who did not find fully-developed cysts as a definitive evidence of sporogenic development. Maier et al. (2000) reported a broad host range of *M. ectocarpii* which might also be due to the fact that the sporangial part of the life cycle appears to be more generalist than the sporogenic part of the phytomyxid life cycle (Neuhauser et al. 2014), but their list of hosts does not include any close relative of *Durvillaea spp*. Therefore, we have only data on the two complementary stages of the life cycle of the two *Maullinia* species, not permitting any direct morphological comparison between the two of them. The two *Maullinia* species were discovered in the same geographical region but in notably different habitats. *M. ectocarpii* was found at the Cariquilda river mouth (Maullin town) in a sandy, shallow and sheltered estuarine environment (ca. 5 m depth), parasitizing *E. siliculosus* that itself is an epiphyte of the red alga *Gracilaria chilensis,* which has been farmed there for decades (Westermeier et al. 1991). *Durvillaea* spp., on the other hand, live in the exposed rocky intertidal (Westermeier et al. 1994), and in our case it was found 25 linear km southward from the type locality of *M. ectocarpii* in Mar Brava, Chiloe. In phylogenies based on a comprehensive taxon sampling of phytomyxids and Rhizaria (Figs 4, 5) the two species form consistently separated clades. Support values are high and distances between the two species are similar or even larger than those between other accepted closely-related species. Also when looking into the individual sequences, there are consistent differences in the rDNA. *M. braseltonii* isolates from the Atlantic Ocean (Falkland Islands) and the Pacific Ocean (Chiloe Island, Central Chile) are more similar (99-100% sequence similarity) to each other than sequences of *M. braseltonii* from Chiloe Island to *M. ectocarpii* in Maullín (88% in the 18S-5.8S-28S sequence). Combined with the morphological features which clearly place the parasite within the Phytomyxea and the molecular phylogenies there is enough evidence for the two organisms being separate species belonging to the same genus.

There have been literature reports of *M. braseltonii* before, but none of them has formally described the species*. M. braseltonii* has been found in *Durvillaea* beds along the Chilean coast and on the Falkland Islands. A parasite resembling *M*. *braseltonii* was reported from Australia (Jahnke 1978) and Mcquarie Island (Ricker 1987), but we could not confirm whether it belongs to the same species, as biological material for comparisons is not available. On the other hand, some of the observations made on the Chilean coast by Aguilera et al. (1988) and by Goecke et al. (2012) contrast our punctual observations in the field. We found that the galls are mainly formed on adult *Durvillaea* fronds during winter months. Instead, Aguilera et al. (1988) and Goecke et al. (2012) for Chilean (continental) *D. antarctica* have not found differences in the prevalence and seasonal appearance of the parasite. Until now, no influence of *Maullinia* infection on the reproductive phenology (based on prevalence of reproductive fronds) of *Durvillaea* spp. has been observed (Aguilera et al. 1988). But given the size and difficulties to access and microscopically screen large numbers of infected individuals in the field this cannot be categorically ruled out. Indeed, consequences on the reproductive cells of *Macrocystis* gametophytes and ectocarpalean seaweeds were observed for infections with *M. ectocarpii* (Maier et al. 2000). The continuous presence for more than 25 years of *Maullinia* in bull kelp populations on the Chilean coast, its wide presence in the southern hemisphere and the lack of reports on devastating effects of infections suggest a balanced relationship between *Durvillaea* spp. and *M. braseltonii*. Currently, the disease has been reported mainly in bull kelp stocks in southern Chile populations (Aguilera et al. 1988; Goecke et al. 2012), and our current knowledge of its distribution coincides with the southern boundary of the continental clade of *Durvillaea* (sensu Fraser et al. 2010b). This allows us to speculate that there can be a link between the susceptibility to *M. braseltonii* of *Durvillaea-*clade/subspecies.

Several plant-associated phytomyxids are well known because they cause significant economic damage (Dixon 2009; Kanyuka et al. 2003; Santala et al. 2010). *Durvillaea* spp. have an increasing commercial potential. Landings in Chile alone surpassed 8,000 t in 2014 (Sernapesca 2015), growing more than four times over the last ten years which is leading to overexploitation problems in Central Chile. For the food industry, a diseased *Durvillaea*-crop is undesirable since galls do not present the organoleptic quality required (e.g. colouration, texture) and stocks with tumours are often rejected by processors (Murúa, personal observation). Currently, *Durvillaea* harvesting therefore focuses on healthy individuals, which are either collected from drifted stocks or harvested by completely removing them from the substratum. It is unknown how this selective fishery pressure based only on healthy individuals would be affecting *Durvillaea* stocks in the near future.

The galls appear to change the elasticity and stability of infected *Durvillaea* fronds, leading to an increased likelihood of breaking/shearing of the fronds. A large number of diseased kelps was seen washed upon the beach. Increased rupture of *Durvillaea* fronds on the one hand impacts on the individual kelp bed by reducing shelter/changing the currents/weakening the site. But on the other hand increased floating – combined with the ability of *Durvillaea* to stay alive when detached – can also lead to a wider distribution of bull kelp as a species and increase the genetic pool available for genetic recombination (as by natural detachment as suggested by Fraser et al. 2010a). Whether or not this increased potential for dispersal influences the gene pool and biodiversity of *Durvillaea* spp. is not known. But it is likely that this process can widely spread the parasite. Overall it will be important to learn more about the epidemiology, the distribution and the impact of *M. braseltonii* on bull kelp and other brown algae.

## Methods

### Field sampling

During 2013–2016, fresh epibiont-free samples of bull kelp were collected at Mar Brava, Chiloe Island (41° 52′ S, 74° 01′ W Southern Chile) (Feb, Jul, Nov 2015 and Aug 2016), Sea Lion Island (52° 26′ S, 59° 05′ W) and Stanley Harbour (51° 41′ S, 57° 49′ W) in the Falkland Islands (Nov 2013) (Supplementary Material Fig. S1). All samples were characterized by the presence of prominent yellow hypertrophies (warts and galls, Fig. 1A). Once in the laboratory, algal tissues were inspected microscopically to ensure the presence of *Maullinia*. Positive samples were stored in i) CTAB for later DNA extractions (Gachon et al. 2009) and ii) 4% paraformaldehyde (PFA) in Provasoli-enriched seawater (PES, Starr and Zeikus 1993) for microscopy. DNA from *M. braseltonii* from Central Chile (Coliumo bay, 36° 52′ S, 72° 95′ W) collected in 2011 by Goecke et al. (2012) was used as well.

### Wax-paraffin embedded samples and light microscopy

After fixation in 4% PFA (prepared with fresh seawater), galls were dehydrated using an ascending series of ethanol (70% and 95% for 2 hrs. and three series of 100%, 3 hrs. each) and defatted/cleared using a 1:1 xylene:chloroform solution for three times 1 h. Subsequently, samples were wax-infiltrated by two baths in wax of 3 h each at room temperature. The final blocks were cut to 10 µm on a Leica RM2125RT microtome and stained with toluidine blue (0.05%) for 15 s. Micrographs were obtained at magnifications of 20× and 40× on an EVOS XL Cell Imaging System (Thermofisher) and x63 on a Axio imager D2™ microscope (Zeiss) coupled with a digital camera (AxioCam HRc, Zeiss). Images were taken with settings adjusted automatically depending on the sample and magnification.

### Fluorescence microscopy

Sections were made manually from material stored in 4% PFA by cutting galls with a sterile surgical blade to approx. 20–40 µm thickness. Sections were stained in i) propidium iodide (1 µg ml^−1^) and incubated in the dark for 15–30 min. Samples were mounted in Slowfade (Invitrogen) in order to increase the stability of the fluorescent dye. Images were taken using a Zeiss confocal LSM 710 microscope with laser excitations of 488 nm, 568 nm and 647 nm and a detection window of 570–630 nm.

A subset of samples from Mar Brava were fixed in 4% PFA and dehydrated with an ascendant series of ethanol (50%, 80%, 96%). These samples were cut by hand to approx. 40–50 µm thickness. Probes Pl_LSU_3690 and Pl_LSU_2313 described by Schwelm et al. (2016) were used for fluorescence in situ hybridisation (FISH), and subsequent hybridisation followed the protocol by the same authors. Samples were analysed using a Leica TCS SP5 II confocal microscope using excitation wavelengths of 405 nm, 488 nm and 514/561 nm and images were recorded using the appropriate emission spectra for DAPI and FAM using the sequential scanning mode.

### Transmission electron microscopy

For transmission electron microscopy we slightly modified the protocol from Sekimoto et al. (2008). The biomass was fixed in a solution of 2.5% glutaraldehyde, 0.1 M cacodylate buffer (pH 7.4), 0.5% caffeine, 0.1% CaCl_2_ and 3% NaCl in Provasoli-enriched seawater (PES) for a couple of days, and then washed three times with 0.1 M cacodylate buffer (pH 7.4), 0.1% CaCl_2_ and 3% NaCl in PES. Afterwards, this material was fixed in 1% OsO_4_ and washed once with a OsO_4_ buffer. Uranyl acetate solution (2% in distilled water) was applied once for 1 hour. Subsequent dehydration was carried out by acetone series (15 minutes at 10%, 20%, 30%, 40%, 50%, 60%, 70%, 80%, 90% and three series of 100%, the last one overnight). Infiltration with Spurr’s resin was subsequently performed through incubating the tissue specimens in a series of differing ratios with acetone (Acetone:Spurr’s: 7:1, 3:1, 1:1, 1:3, 1:6; 12 h each) until finally being incubated in 100% Spurr’s resin and polymerized at 60°–70 °C. Samples were then sectioned at 90 nm using an ultramicrotome (Leica UC6) and placed on copper grids before being contrast stained with lead citrate (3%). Sections were imaged using a JEM-1400 Plus (JEOL) TEM with an AMT UltraVue camera, available at the Aberdeen microscopy facility.

### DNA extraction and sequencing

About 40 mg DW per sample were used for DNA extractions. They were performed using the DNA purification kit developed by Thermo Scientific® (GeneJET™ Plant Genomic DNA Purification Kit), complemented by the application of 700 µl CTAB buffer in the grinding process. Polymerase chain reactions (PCR) were carried out to amplify afragment of the 18S nuclear ribosomal DNA, using the primer pair Mau2F and Mau9R and procedures provided by Goecke et al. (2012), but using an annealing temperature at 64 °C. For the 18S-5.8S-28S region amplifications, we modified Schwelm et al. (2016) protocol. A First PCR was carried out using primers V7fmix (equimolar mixture of V7fPhag and V7fPlas) and 28S4R (touchdown PCR, annealing temperature: from 62 °C to 54 °C). In a second PCR, primers C9fmix (equimolar mixture of C9fPhag and C9fPlas) and D14rphyt were used (annealing temperature: 65 °C). With this nested product a third PCR was carried out using C9fmix and Cdrplas (annealing temperature as before). The final products were purified using the GeneJet™ nucleic acid purification kit.

PCR products were Sanger-sequenced (Source bioscience, United Kingdom) with the primers aforementioned (Mau2F and Mau9R for 18S, C9fPlas and D14rphyt for 18S-5.8S-28S), aligned and consensus sequences were generated. The resulting sequences were aligned to a representative selection of 18S and combined 18S-5.8S-28S rDNA sequences of Phytomyxea, Endomyxa, Alveolata and Stramenopiles containing a total of 42 and 30 sequences respectively, three of which are new in this study. Sequences were initially aligned using MAFFT (Katoh and Standley 2013) implemented in Geneious (Kearse et al. 2012; R9.1.5) using the default settings and a 1PAM/k = 2 scoring matrix. The alignment was subsequently improved manually to take the highly variable regions into account. Trees were generated using three different models: PhyML (Guindon et al. 2010), RAxML (Stamatakis 2014) and MrBayes v 3.1.2 (Ronquist et al. 2012) as implemented in the Geneious software. The exact settings used to calculate the trees were estimated from the data and are given with the trees (Figs 5, 6; Supplementary Material Figs. S2-S4). Alignments were deposited at figshare.org DOI: 10.6084/m9.figshare.5065507.

## Supplementary Material

Appendix A

## Figures and Tables

**Table 1:** Median size of galls, plasmodia and resting spores. The individual values can be found in the Supplement.

	Length [Median]	Width [Median]	Length to width ratio [Median]	Min length	Min width	Max length	Max width
Spores [μm], n = 10	3.2	2.7	1.18	2.6	2.1	4.0	3.3
Plasmodia [μm] n = 120	61	37	1.8	27	18	119	73
Galls [mm] n = 38	45.92	32.71		9	9	97	67

## References

[R1] Aguilera M, Rivera PJ, Westermeier R (1988). The presence of Plasmodiophorales in plants of *Durvillaea antarctica* (Cham.) Hariot (Phaeophyta, Durvilleacece) in Southern Chile. Gayana Bot.

[R2] Bulman S, Braselton JP, McLaughlin DJ, Spatafora JW (2014). Rhizaria: Phytomyxea. The Mycota VII, Part A, Systematics and Evolution.

[R3] Bulman SR, Kühn SF, Marshall JW, Schnepf E (2001). A phylogenetic analysis of the SSU rRNA from members of the Plasmodiophorida and Phagomyxida. Protist.

[R4] Castilla J, Bustamante R (1989). Human exclusion from rocky intertidal of Las Cruces, central Chile: effects on *Durvillaea antarctica* (Phaeophyta, Durvilleales). Mar Ecol Prog Ser.

[R5] Castilla JC, Campo MA, Bustamante RH (2007). Recovery of *Durvillaea antarctica* (Durvilleales) inside and outside Las Cruces Marine Reserve, Chile. Ecol Appl.

[R6] Dillehay TD, Ramirez C, Pino M, Collins MB, Rossen J, Pino-Navarro JD (2008). Monte Verde: seaweed, food, medicine, and the peopling of South America. Science.

[R7] Dixon GR (2009). The occurrence and economic impact of *Plasmodiophora brassicae* and clubroot disease. J Plant Growth Regul.

[R8] Fraser CI, Nikula R, Waters JM (2011). Oceanic rafting by a coastal community. Proc R Soc B Biol Sci.

[R9] Fraser CI, Thiel M, Spencer HG, Waters JM (2010a). Contemporary habitat discontinuity and historic glacial ice drive genetic divergence in Chilean kelp. BMC Evol Biol.

[R10] Fraser CI, Winter DJ, Spencer HG, Waters JM (2010b). Multigene phylogeny of the southern bull-kelp genus *Durvillaea* (Phaeophyceae: Fucales). Mol Phylogenet Evol.

[R11] Gachon CMM, Sime-Ngando T, Strittmatter M, Chambouvet A, Kim GH (2010). Algal diseases: Spotlight on a black box. Trends Plant Sci.

[R12] Gachon CMM, Strittmatter M, Müller DG, Kleinteich J, Küpper FC (2009). Detection of differential host susceptibility to the marine oomycete pathogen *Eurychasma dicksonii* by real-time PCR: Not all algae are equal. Appl Environ Microbiol.

[R13] Goecke F, Labes A, Wiese J, Imhoff JF (2010). Review chemical interactions between marine macroalgae and bacteria. Mar Ecol Prog Ser.

[R14] Goecke F, Wiese J, Núñez A, Labes A, Imhoff JF, Neuhauser S (2012). A novel phytomyxean parasite associated with galls on the bull-kelp *Durvillaea antarctica* (Chamisso) Hariot. PLoS ONE.

[R15] Graiff A, Karsten U, Meyer S, Pfender D, Tala F, Thiel M (2013). Seasonal variation in floating persistence of detached *Durvillaea antarctica* (Chamisso) Hariot thalli. Bot Mar.

[R16] Guindon S, Dufayard JF, Lefort V, Anisimova M, Hordijk W, Gascuel O (2010). New algorithms and methods to estimate Maximum-Likelihood phylogenies: Assessing the performance of PhyML 3. 0. Syst Biol.

[R17] Guiry MD, Guiry GM (2016). Durvillaea [WWW Document]. AlgaeBase. World-wide Electron Publ Natl Univ, Ireland, Galway.

[R18] Jahnke R (1978). A study of gall diseased laminae of the marine brown alga *Durvillaea potatorum* (Labilladiere) Areschoug. La Trobe University.

[R19] Kanyuka K, Ward E, Adams MJ (2003). *Polymyxa graminis* and the cereal viruses it transmits: A research challenge. Mol Plant Pathol.

[R20] Katoh K, Standley DM (2013). MAFFT multiple sequence alignment software version 7: Improvements in performance and usability. Mol Biol Evol.

[R21] Kearse M, Moir R, Wilson A, Stones-Havas S, Cheung M, Sturrock S, Buxton S, Cooper A, Markowitz S, Duran C, Thierer T (2012). Geneious Basic: An integrated and extendable desktop software platform for the organization and analysis of sequence data. Bioinformatics.

[R22] López BA, Macaya EC, Tala F, Tellier F, Thiel M (2017). The variable routes of rafting: stranding dynamics of floating bull kelp *Durvillaea antarctica* (Fucales, Phaeophyceae) on beaches in the SE Pacific. J Phycol.

[R23] Loureiro R, Gachon CMM, Rebours C (2015). Seaweed cultivation: potential and challenges of crop domestication at an unprecedented pace. New Phytol.

[R24] Maier I, Parodi E, Westermeier R, Müller DG (2000). *Maullinia ectocarpii* gen. et sp. nov. (Plasmodiophorea), an intracellular parasite in *Ectocarpus siliculosus* (Ectocarpales, Phaeophyceae) and other filamentous brown algae. Protist.

[R25] Miller C (1958). Morphology and cytology of the zoosporangia and cystosori of *Sorosphaera veronicae*. J Elisha Mitchell Sci Soc.

[R26] Müller DG, Küpper FC, Küpper H (1999). Infection experiments reveal broad host ranges of *Eurychasma dicksonii* (Oomycota) and *Chytridium polysiphoniae* (Chytridiomycota), two eukaryotic parasites in marine brown algae (Phaeophyceae). Phycol Res.

[R27] Neuhauser S, Kirchmair M, Gleason FH (2011). Ecological roles of the parasitic phytomyxids (plasmodiophorids) in marine ecosystems - A review. Mar Freshw Res.

[R28] Neuhauser S, Kirchmair M, Bulman S, Bass D (2014). Cross-kingdom host shifts of phytomyxid parasites. BMC Evol Biol.

[R29] Parodi ER, Cáceres EJ, Westermeier R, Müller DG (2010). Secondary zoospores in the algal endoparasite *Maullinia ectocarpii* (Plasmodiophoromycota). Biocell.

[R30] Ricker RW (1987). Taxonomy and biogeography of Macquarie Island seaweeds. British Museum (Natural History), London.

[R31] Ronquist F, Teslenko M, Van Der Mark P, Ayres DL, Darling A, Höhna S, Larget B, Liu L, Suchard MA, Huelsenbeck JP (2012). MrBayes 3. 2: Efficient bayesian phylogenetic inference and model choice across a large model space. Syst Biol.

[R32] Santala J, Samuilova O, Hannukkala A, Latvala S, Kortemaa H, Beuch U, Kvarnheden A, Persson P, Topp K, Ørstad K, Spetz C (2010). Detection, distribution and control of Potato mop-top virus, a soil-borne virus, in northern Europe. Ann Appl Biol.

[R33] Sawabe T, Makino H, Tatsumi M, Nakano K, Tajima K, Iqbal MM, Yumoto I, Ezura Y, Christen R (1998). *Pseudoalteromonas bacteriolytica* sp. nov., a marine bacterium that is the causative agent of red spot disease of *Laminaria japonica*. Int J Syst Bacteriol.

[R34] Schiel DR, Nelson WA (1990). The harvesting of macroalgae in New Zealand. Hydrobiologia.

[R35] Schnepf E, Kühn SF, Bulman S (2000). *Phagomyxa bellerocheae* sp. nov. and *Phagomyxa odontellae* sp. nov., Plasmodiophoromycetes feeding on marine diatoms. Helgol Mar Res.

[R36] Schroeder DC (2015). More to Phaeovirus infections than first meets the eye. Perspect Phycol.

[R37] Schwelm A, Berney C, Dixelius C, Bass D, Neuhauser S (2016). The large subunit rDNA sequence of *Plasmodiophora brassicae* does not contain intra-species polymorphism. Protist.

[R38] Sekimoto S, Beakes GW, Gachon CMM, Müller DG, Küpper FC, Honda D (2008). The development, ultrastructural cytology, and molecular phylogeny of the basal oomycete *Eurychasma dicksonii* infecting the filamentous phaeophyte algae *Ectocarpus siliculosus* and *Pylaiella littoralis*. Protist.

[R39] Sernapesca (2015). Desembarque artesanal por region [WWW Document]. Anuario Estadistico de Pesca 2014.

[R40] South GR (1974). *Herpodiscus* gen. nov. and *Herpodiscus durvilleae* (Lindauer) comb. nov., a parasite of *Durvillea antarctica* (Chamisso) hariot endemic to New Zealand. J R Soc New Zeal.

[R41] Stamatakis A (2014). RAxML version 8: A tool for phylogenetic analysis and post-analysis of large phylogenies. Bioinformatics.

[R42] Starr RC, Zeikus JA (1993). UTEX-The Culture Collection of algae at the University of Texas at Austin 1993 List of cultures. J Phycol.

[R43] Taylor DI, Schiel DR (2005). Self-replacement and community modification by the southern bull kelp *Durvillaea antarctica*. Mar Ecol Prog Ser.

[R44] Westermeier R, Müller DG, Gómez I, Rivera P, Wenzel H (1994). Population biology of *Durvillaea antarctica* and *Lessonia nigrescens* (Phaeophyta) on the rocky shores of Southern Chile. Mar Ecol Ser.

[R45] Westermeier R, Rivera P, Gomez I (1991). Cultivo de *Gracilaria chilensis* Bird, McLachlan y Oliveira, en la zona intermareal y submareal del Estuario Cariquilda, Maullín, Chile. Rev Chil Hist Nat.

