# Supplementary material for: Maullinia braseltonii sp. nov. (Rhizaria, Phytomyxea, Phagomyxida): A Cyst-forming Parasite of the Bull Kelp Durvillaea spp. (Stramenopila, Phaeophyceae, Fucales)
Source: Protist. Author manuscript; Available in PMC 2017 Nov 6. (PMC5673062; doi:10.1016/j.protis.2017.07.001)
Supplement: Appendix A [file NIHMS73520-supplement-Appendix_A.pdf]

Supplementary Information

Maullinia braseltonii sp. nov. (Rhizaria, Phytomyxea, Phagomyxida): A cyst-forming parasite of the bull kelp *Durvillaea* spp. (Stramenopila, Phaeophyceae, Fucales)

Pedro Murúa[1, 2, 3], Franz Goecke[4], Renato Westermeier[3], Pieter van West[2],

Frithjof C. Küpper[1], Sigrid Neuhauser[5].

Index of Items:

Supplementary Figure 1. Geographic origin of samples. p2

Supplementary Figure 2: 18S-rDNA PhyML tree supplementing Fig. 5. p3

Supplementary Figure 3: 18S-rDNA RAxML tree supplementing Fig. 5. p4

Supplementary Figure 4: Partial-18S-5.8S-partial-28S rDNA RAxML tree supplementing Fig. 6. p5

Supplementary Table 1: Size of the galls caused by *M. braseltonii* on the fronds of *D. antarctica* in Mar Brava. p6

Supplementary Table 2: Size of plasmodia formed by *M. braseltonii*. p7-8

Supplementary Table 3: Size of resting spores formed by *M. braseltonii*. p9-10

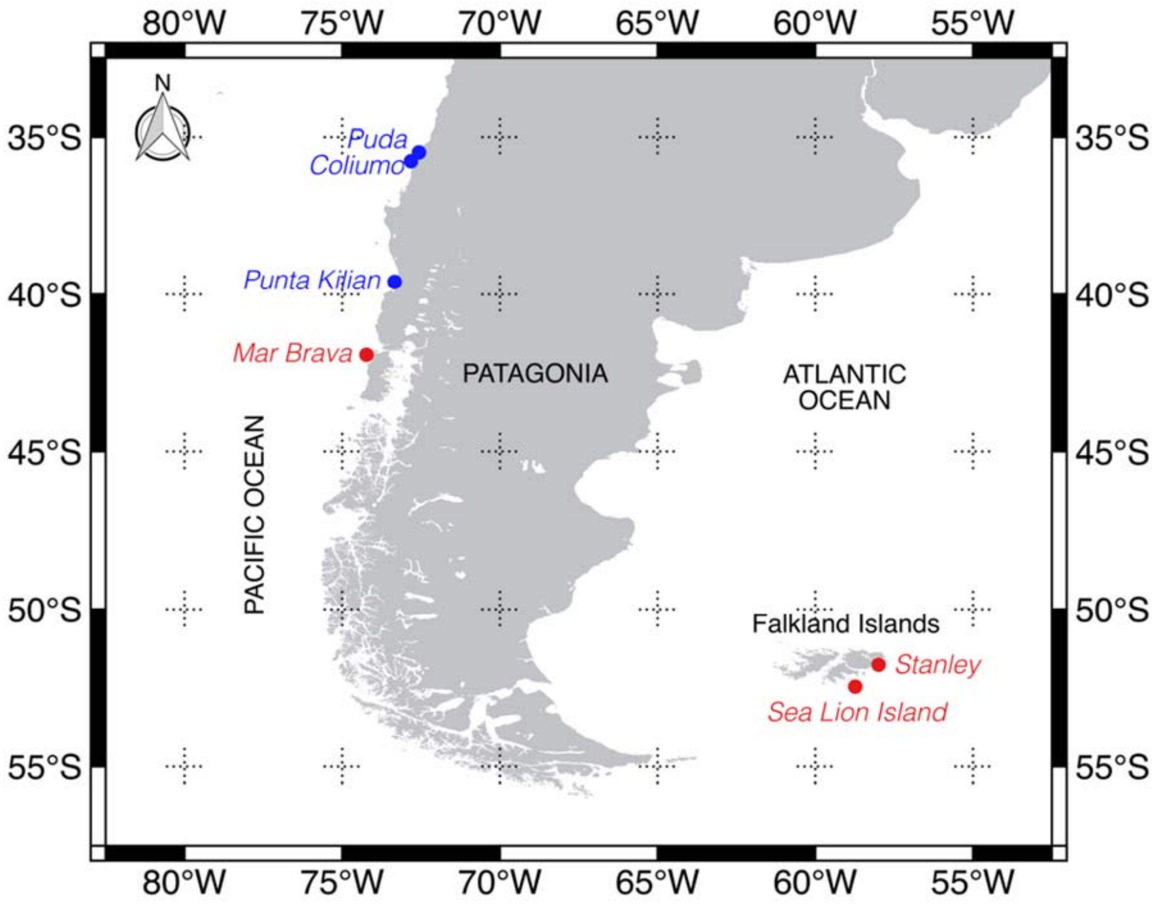

Supplementary Figure 1. Geographic origin of samples. Localities in the South-eastern Pacific and the Atlantic where our biological material and/or sequences came from (red dots), which constitute new records for Maullinia braseltonii. Old records of the species are indicated with blue dots.

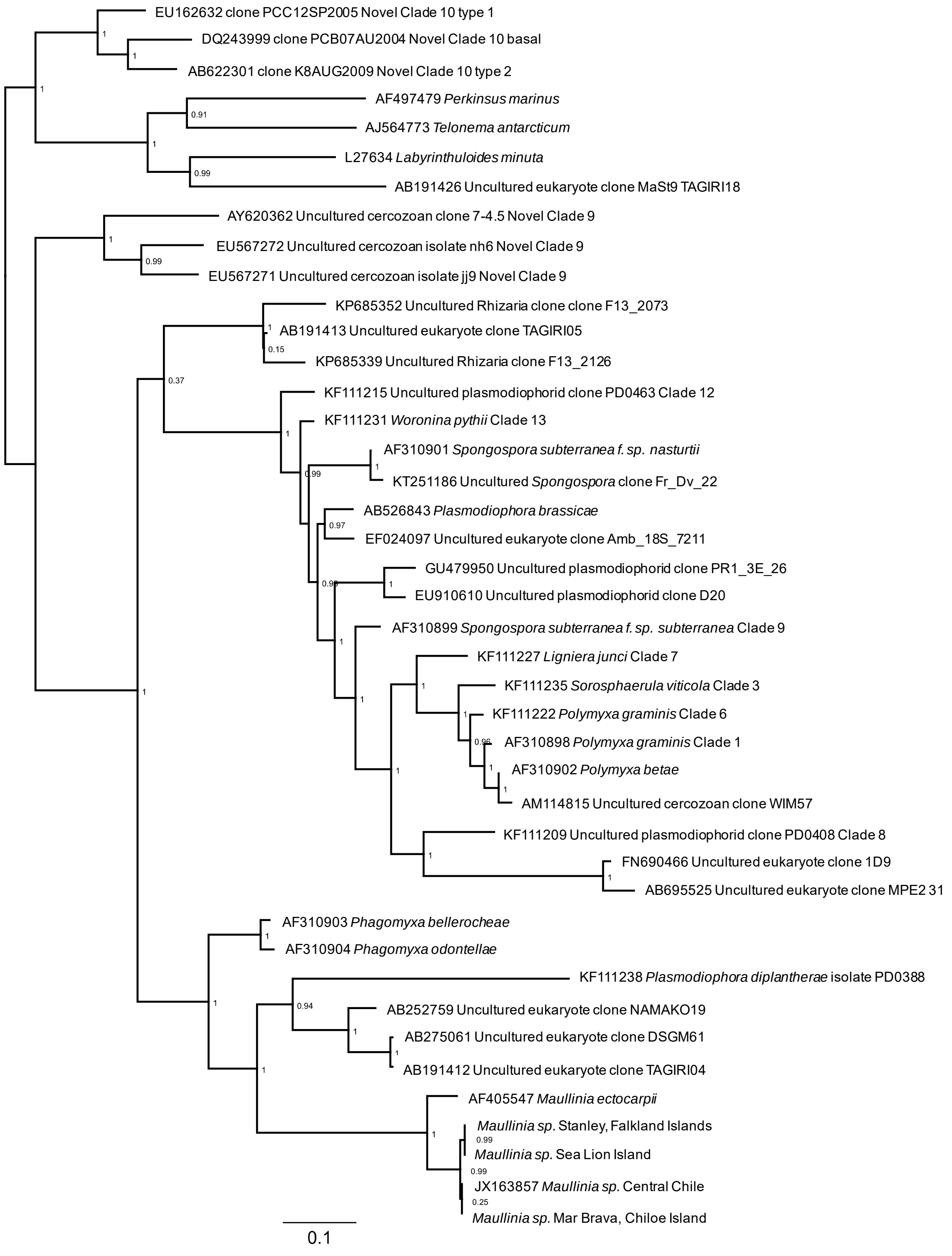

Supplementary Figure 2: 18S-rDNA PhyML tree supplementing Fig. 5. 43 sequences and 1002 positions. Model used was JC 69 with searching for Best of NNIs and SPRs. χ^2 support values are given. All major branches are well supported and the separation into M. ectocarpii and M. braseltonii has a high support value.

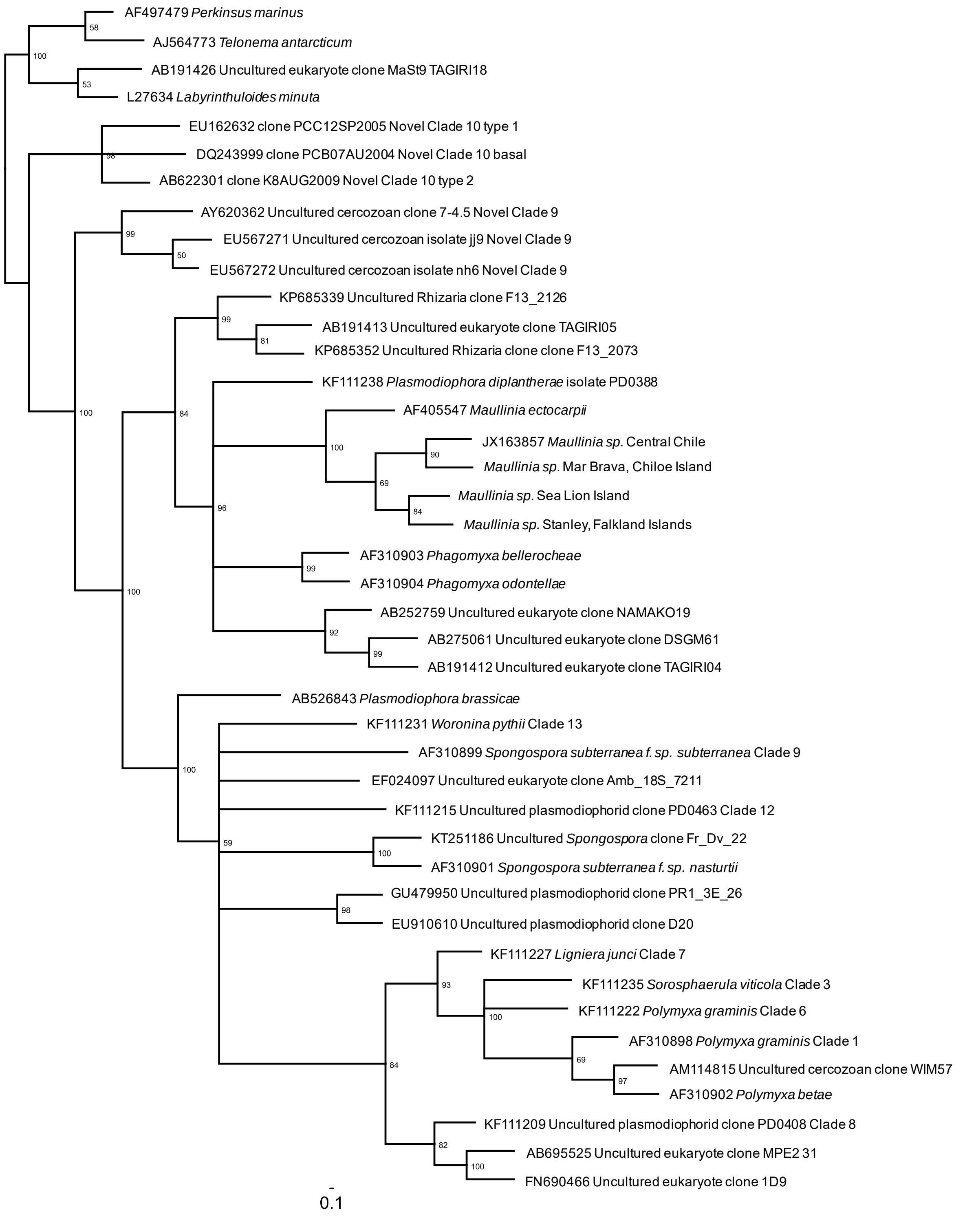

Supplementary Figure 3: 18S-rDNA RAxML tree supplementing Fig. 5. 43 sequences and 1002 positions. Tree was generated using the GTR Gamma I model with 100 rapid bootstrapping replicates. The displayed consensus tree was generated with using the Consensus tree builder implemented in Geneious using a support threshold of 50% and a burn in of 10% of the trees. Support for the *M. braseltonii* branch is rather low with a bootstrap vaule of 69, however, the support values for two clades within M. braseltonii is high compared to the PhyML and MrBayes trees, explain the low support of the main branch of the species.

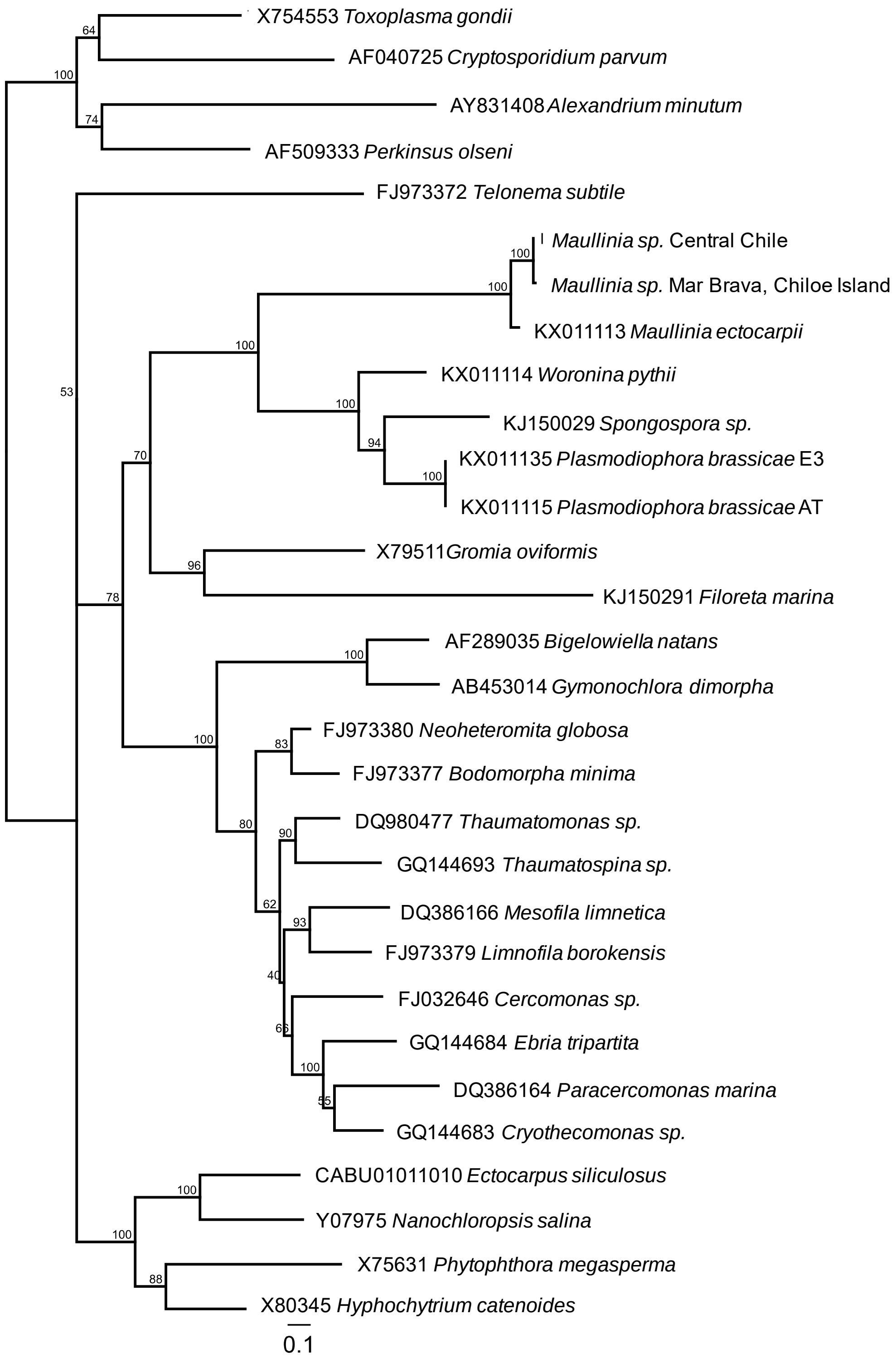

Supplementary Figure 4: partial-18S-5.8S-partial-28S rDNA RAxML tree supplementing Fig. 6. 30 sequences and 1767 positions (ITS1, ITS2 were excluded from the analyses). Tree was generated using GTR+CAT+I with 100 bootsrap replicates and a search for the best scoring ML tree.

Supplementary Table 1: Size of the galls caused by *M. braseltonii* on the fronds of *D. antarctica* in Mar Brava (n=38). Galls were measured *in situ* using a regular Vernier caliper. A speciemen number was assigned to each analysed frond and all galls on each individual were measured across the two broadest planes.

Gall size (cm)					
Specimen	Length	Width	Specimen	Length	Width
1	5.3	2	3	5.2	3.8
1	2.3	2	4	9.7	6.7
1	2.2	1.8	4	8.2	6.1
1	1.9	0.9	5	3.3	3.5
1	1.8	1.6	5	1.9	1.8
1	2.1	1.9	6	9.1	5.3
1	2.1	1	6	7.2	5.3
1	2	1.7	6	4.1	3.1
1	2	1.9	6	8.8	5.9
1	2.2	1	6	3.1	2
1	2	1.1	6	5.2	4.1
1	0.9	0.9	6	5.5	5
1	1.9	1.9	6	8	6.5
1	3.9	2.8	6	6.1	3.3
1	2.2	1	6	5.4	5.1
1	2.3	1.1	6	6.9	5
2	8.5	4.9	6	8.4	5.2
2	7.4	4.1	7	3.9	3.7
3	7.2	5.9	8	4.3	3.4
			Median	4	3.2
			Average	4.59	3.27
			Max	9.7	6.7
			Min	0.9	0.9

Supplementary Table 2: Size of the plasmodia formed by *M. braseltonii* (n= 120). Plasmodium dimensions were calculated *in silico* from microscopic images (Axiocam HRc), using the image analysis software FIJI.

Plasmodia size (um)					
Length	Width	Ratio	Length	Width	Ratio
44	37	1.189189189	82	49	1.673469388
68	61	1.114754098	119	71	1.676056338
49	45	1.088888889	52	31	1.677419355
34	33	1.03030303	59	35	1.685714286
44	43	1.023255814	71	42	1.69047619
35	35	1	73	43	1.697674419
27	27	1	67	39	1.717948718
39	38	1.026315789	45	26	1.730769231
38	37	1.027027027	66	38	1.736842105
40	38	1.052631579	91	52	1.75
32	30	1.066666667	69	39	1.769230769
78	73	1.068493151	55	31	1.774193548
47	42	1.119047619	73	41	1.780487805
59	52	1.134615385	67	37	1.810810811
56	49	1.142857143	42	23	1.826086957
50	43	1.162790698	66	36	1.833333333
43	36	1.194444444	72	39	1.846153846
47	39	1.205128205	76	41	1.853658537
56	46	1.217391304	71	38	1.868421053
50	41	1.219512195	64	34	1.882352941
42	34	1.235294118	64	34	1.882352941
78	63	1.238095238	81	43	1.88372093
57	46	1.239130435	61	32	1.90625
64	51	1.254901961	46	24	1.916666667
44	35	1.257142857	71	37	1.918918919
57	45	1.266666667	81	42	1.928571429
54	42	1.285714286	81	42	1.928571429
63	49	1.285714286	60	31	1.935483871
44	34	1.294117647	66	34	1.941176471
66	51	1.294117647	68	35	1.942857143
60	45	1.333333333	43	22	1.954545455
53	39	1.358974359	43	22	1.954545455
34	25	1.36	45	23	1.956521739
44	32	1.375	60	30	2
72	52	1.384615385	66	33	2
60	43	1.395348837	75	37	2.027027027
28	20	1.4	53	26	2.038461538
56	40	1.4	64	31	2.064516129
35	25	1.4	58	28	2.071428571
62	44	1.409090909	73	35	2.085714286
62	44	1.409090909	57	27	2.111111111
58	40	1.45	49	23	2.130434783
41	28	1.464285714	54	25	2.16

Plasmodia size (um)					
Length	Width	Ratio	Length	Width	Ratio
79	53	1.490566038	54	25	2.16
90	60	1.5	67	30	2.233333333
66	44	1.5	65	29	2.24137931
65	43	1.511627907	48	21	2.285714286
47	31	1.516129032	87	38	2.289473684
61	40	1.525	63	27	2.333333333
46	30	1.533333333	97	41	2.365853659
54	35	1.542857143	95	40	2.375
65	42	1.547619048	88	37	2.378378378
48	31	1.548387097	70	29	2.413793103
85	54	1.574074074	77	31	2.483870968
80	50	1.6	72	26	2.769230769
54	33	1.636363636	55	18	3.055555556
54	33	1.636363636	83	25	3.32
64	39	1.641025641	84	24	3.5
87	53	1.641509434	104	28	3.714285714
90	54	1.666666667	109	28	3.892857143
		Median	61	37	1.67
		Average	61.85	37.46	1.72
		Standard deviation	17.18	10.29	0.54
		Max	119	73	3.89
		Min	27	18	1

Supplementary Table 3: Size of resting spores formed by *M. braseltonii* (n= 110). Resting spore dimensions were calculated *in silico* from microscopic images (Axiocam HRc), using the image analysis software FIJI.

Spore size (um)					
Length	Width	Ratio	Length	Width	Ratio
3.592	2.395	1.499791232	3.172	2.695	1.176994434
3.464	2.322	1.491817399	3.077	2.615	1.17667304
3.355	2.283	1.4695576	3.105	2.643	1.174801362
3.205	2.183	1.468163078	3.243	2.762	1.174149167
3.365	2.293	1.467509812	2.577	2.195	1.174031891
3.452	2.364	1.460236887	2.741	2.357	1.162918965
3.562	2.466	1.444444444	2.832	2.436	1.162561576
3.385	2.357	1.436147645	2.695	2.322	1.160637382
3.205	2.239	1.431442608	2.695	2.322	1.160637382
3.631	2.538	1.430654058	3.172	2.741	1.157241883
3.649	2.554	1.428739233	3.421	2.96	1.155743243
3.122	2.195	1.422323462	2.812	2.439	1.152931529
3.387	2.409	1.405977584	3.824	3.327	1.149383829
2.982	2.122	1.40527804	3.462	3.039	1.139190523
2.952	2.114	1.39640492	3.276	2.881	1.137105172
3.471	2.489	1.394535958	3.001	2.643	1.135452138
3.527	2.545	1.385854617	3.427	3.039	1.127673577
3.452	2.564	1.346333853	3.046	2.717	1.121089437
3.328	2.529	1.315935152	3.343	2.982	1.121059691
3.215	2.449	1.312780727	3.309	2.952	1.120934959
2.924	2.239	1.305940152	3.44	3.08	1.116883117
3.96	3.039	1.303060217	3.718	3.333	1.115511551
3.286	2.525	1.301386139	3.452	3.119	1.106764989
3.365	2.593	1.297724643	2.924	2.643	1.106318577
3.464	2.695	1.285343228	3.365	3.047	1.104364949
3.228	2.519	1.281460897	2.564	2.322	1.1042205
2.867	2.24	1.279910714	3.599	3.262	1.103310852
3.45	2.72	1.268382353	3.004	2.726	1.101980924
2.926	2.311	1.266118563	3.373	3.08	1.09512987
3.491	2.776	1.257564841	3.088	2.821	1.094647288
3.239	2.577	1.256887854	2.695	2.466	1.092862936
3.058	2.436	1.255336617	3.053	2.8	1.090357143
3.373	2.695	1.251576994	3.343	3.077	1.086447839
3.048	2.436	1.251231527	3.336	3.077	1.084172896
3.205	2.564	1.25	3.205	2.96	1.08277027
3.205	2.567	1.248539151	3.421	3.172	1.078499369
2.881	2.311	1.246646473	2.643	2.466	1.071776156
3.173	2.554	1.242364918	3.048	2.847	1.070600632
3.215	2.593	1.239876591	3.333	3.121	1.067926946
3.52	2.847	1.236389182	3.08	2.892	1.065006916
3.774	3.053	1.236161153	3.269	3.082	1.060674886
3.101	2.525	1.228118812	3.246	3.077	1.054923627
3.671	2.99	1.227759197	2.704	2.577	1.049282111

Spore size (um)					
Length	Width	Ratio	Length	Width	Ratio
3.004	2.449	1.226623111	3.246	3.101	1.04675911
3.018	2.466	1.223844282	3.073	2.949	1.042048152
3.45	2.823	1.222104145	2.8	2.72	1.029411765
3.269	2.692	1.214338782	2.838	2.762	1.027516293
3.592	2.96	1.213513514	3.269	3.185	1.026373626
2.924	2.419	1.208763952	3.026	2.952	1.025067751
3.494	2.901	1.204412272	3.098	3.039	1.019414281
2.867	2.395	1.197077244	3.13	3.088	1.013601036
3.119	2.615	1.192734226	3.08	3.047	1.010830325
3.208	2.695	1.190352505	2.952	2.926	1.008885851
3.537	2.974	1.18930733	3.166	3.166	1
3.488	2.96	1.178378378	2.692	2.692	1
		Median	3.215	2.692	1.177686406
		Average	3.210890909	2.693681818	1.201715563
		Standard deviation	0.28	0.30	0.13
		Max	3.96	3.333	1.499791232
		Min	2.564	2.114	1
